# Ruminal acidosis and the rapid onset of ruminal parakeratosis in a mature dairy cow: a case report

**DOI:** 10.1186/1751-0147-51-39

**Published:** 2009-10-19

**Authors:** Michael A Steele, Ousama AlZahal, Sarah E Hook, Jim Croom, Brian W McBride

**Affiliations:** 1 Department of Animal and Poultry Science, University of Guelph, Guelph, Canada; 2 Department of Poultry Science, North Carolina State University, Raleigh, USA

## Abstract

A mature dairy cow was transitioned from a high forage (100% forage) to a high-grain (79% grain) diet over seven days. Continuous ruminal pH recordings were utilized to diagnose the severity of ruminal acidosis. Additionally, blood and rumen papillae biopsies were collected to describe the structural and functional adaptations of the rumen epithelium. On the final day of the grain challenge, the daily mean ruminal pH was 5.41 ± 0.09 with a minimum of 4.89 and a maximum of 6.31. Ruminal pH was under 5.0 for 130 minutes (2.17 hours) which is characterized as the acute form of ruminal acidosis in cattle. The grain challenge increased blood beta-hydroxybutyrate by 1.8 times and rumen papillae mRNA expression of 3-hydroxy-3-methylglutaryl-coenzyme A synthase by 1.6 times. Ultrastructural and histological adaptations of the rumen epithelium were imaged by scanning electron and light microscopy. Rumen papillae from the high grain diet displayed extensive sloughing of the stratum corneum and compromised cell adhesion as large gaps were apparent between cells throughout the strata. This case report represents a rare documentation of how the rumen epithelium alters its function and structure during the initial stage of acute acidosis.

## Background

In response to the demands for increased feed conversion, cattle, sheep and goat producers rely on rapidly fermentable (high - grain) diets to maximize energy intake. Ruminants fed high - grain diets are at a greater risk of developing ruminal or metabolic acidosis, which may severely compromise gastrointestinal function, feed conversion, and the health and welfare of the animal. The clinical manifestations of this disease in cattle include depressed feed intake and milk production, laminitis, liver abscesses, diarrhea and extensive alterations of rumen microflora populations and their fermentation products [1].

Rumen mucosal damage is another consequence associated with rumen acidosis [2]. The rumen mucosa plays a vital role in whole-animal energy balance through the transport and metabolism of rumen-derived volatile fatty acids (VFAs) [3]. In addition, the rumen epithelium acts as a protective barrier between the rumen environment and portal circulation [4]. Ruminal acidosis has been associated with a high incidence of ruminal wall lesions and the condition of ruminal parakeratosis. The characteristics of ruminal parakeratosis include accumulated layers of keratinized, nucleated squamous epithelial cells and excessive sloughing of the epithelium [5,6].

To date, most reports of rumen mucosa structural and functional alterations from grain-induced ruminal acidosis have been examined after several weeks of adaptation. The objective of this case report was to describe ultrastructural, morphological, and functional adaptations in the rumen mucosa observed in a cow during the early stages of a grain-induced ruminal acidosis.

## Case presentation

A 10-year old, non-lactating Holstein dairy cow, fitted with a rumen cannula, was nutritionally induced to develop ruminal acidosis by increasing the proportion of grain in the diet. All protocols used in the study were approved by the University of Guelph Animal Care Committee. For several years prior to the study, the cow was fed a diet consisting exclusively of hay and supplemental minerals and vitamins. A high forage (HF) diet was fed for one week (days 1 to 7) followed by a transition to a high - grain (HG) diet, from days 8 to 14. The HF diet consisted of 11.0 kg chopped hay with 100 g of supplemental mineral and vitamins (90.6% dry matter, 114 g crude protein/kg dry matter, 600 g neutral detergent fibre/kg dry matter, 173 g non-fibre carbohydrate/kg dry matter, 69 g starch/kg dry matter). The HG diet fed on day 14 consisted of 3.3 kg of chopped hay, 12.5 kg of mixed grains (40% ground wheat, 40% ground barley, 20% ground corn) and 100 g of supplemental mineral and vitamins (88.9% dry matter, 117 g crude protein/kg dry matter, 240 g neutral detergent fibre/kg dry matter, 580 g non-fibre carbohydrate/kg dry matter, 501 g starch/kg dry matter). Throughout the case study, the chopped hay used in each diet was fed in equal allotments daily at 0800 h and 1600 h. From days 8 to 14, equal allotments of mixed grain were fed at 0800 h, 1200 h and 1600 h. Continuous ruminal pH recordings, blood samples and rumen papillae biopsies were collected from the ventral sac on day 7 (HF) and 14 (HG) for assessment.

### Feed intake, ruminal pH, blood metabolite and rumen papillae gene expression

**Figure 1:**
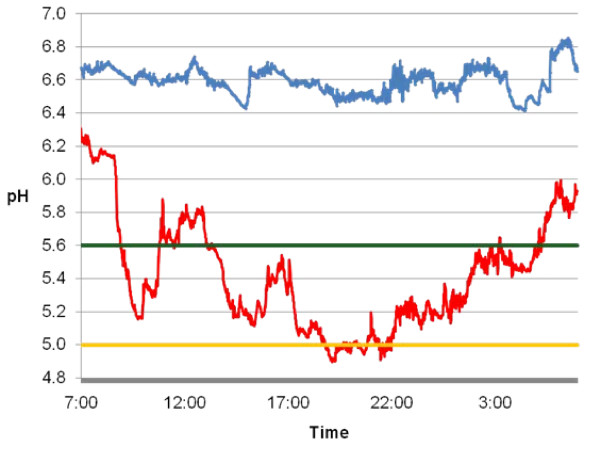
**Continuous rumen pH measurements taken every minute during the high forage (blue; day 7) and high grain (red; day 14) diets**. The green line represents the pH 5.6 threshold and the orange line represents the pH 5.0 threshold for acute ruminal acidosis.

From days 1 to 14 of the study, the cow consumed all dietary components. Immediately after the final day of the protocol, the cow became visibly lethargic and refused feed. Ruminal acidosis was thought to be a likely cause of the clinical signs observed. To confirm ruminal acidosis, continuous ruminal pH measurements were examined to determine the total time per day pH was below 5.6 (sub-acute ruminal acidosis) and 5.0 (acute ruminal acidosis) [2]. Continuous ruminal pH technology and methodology described by AlZahal et al. [7] was used to measure ruminal pH every minute for 24 hours on day 7 (HF) and day 14 (HG). The comparison of pH measurements taken during the HF and HG diets are summarized in Figure 1. The HF diet resulted in a mean ruminal pH of 6.58 ± 0.02 with a daily minimum of 6.42 and a maximum of 6.85. In contrast, the average ruminal pH during the HG was 5.41 ± 0.09 with a minimum of 4.89 and a maximum of 6.31. Ruminal pH was below 5.6 for 1,073 minutes (17.9 hours) and under 5.0 for 130 minutes (2.2 hours) during the HG, therefore reaching pH levels characterized as acute ruminal acidosis [2].

The concentration of beta-hydroxybutyrate (BHBA), non-esterifed fatty acids (NEFA) and glucose in the blood on day 7 (HF) and day 14 (HG) was determined by the Animal Health Laboratory Services Division, University of Guelph, Ontario. Blood was sampled at 0700 h, 1100 h, 1500 h and 1900 h and results from each day were pooled and compared statistically using a paired t-test (*P *< 0.05). Plasma glucose was increased (*P *= 0.02) during the HG diet (4.18 mmol/L) compared to the HF diet (3.73 mmol/L) which can be explained by the greater intake of metabolizable energy (39.2 Mcal/d = 164.1 MJ/d) compared to the HF (19.1 Mcal/d = 79.5 MJ/d). The greatest difference in the blood metabolite analysis was the increase in BHBA (*P *< 0.01) in the HG diet (606.75 μmol/L) versus the HF diet (361.25 μmol/L). Since the NEFA levels did not increase on the HG diet (0.05 mmol/L) versus the HF diet (0.10 mmol/L), the elevated plasma BHBA is hypothesized to be derived from increased ketogenesis in the rumen epithelium.

To examine the expression of a prominent gene involved in ketogenesis, rumen papillae were biopsied from the ventral sac of the rumen via the cannula immediately after the final day of the HF and HG treatments. Rumen papillae biopsies were conducted by the methods of Kelly et al. which noted no carryover effect from repeated rumen papillae biopsies [8]. In brief, the reticulorumen contents were partially evacuated to facilitate the retraction of the ventral sac. Rumen papillae were excised (approximately 100 mg) from similar yet distinct areas of the ventral sac and samples were washed immediately in ice-cold phosphate buffered saline (pH 7.4) ten times prior to freezing in liquid nitrogen. RNA was isolated and quantitative real-time polymerase chain reaction (qRT-PCR) was performed by the methods of Harvatine and Bauman [9]. The expression of 3-hydroxy-3-methylglutaryl-coenzyme A synthase (*HMGCS1*; 5'-AGAGGATCGGCGTGTTTTCTT-3', 5'-CAGACCCTGGTGTGGCATCT-3') was assessed using glyceraldehyde 3-phosphate dehydrogenase (*GAPD*; 5'-TGGAAAGGCCATCACCATCT-3', 5'-CCCACTTGATGTTGGCAG-3') as a house-keeping gene via the Pfaffl method [10]. *HMGCS1 *expression was increased 1.6 times during the acidosis diet which supports the hypothesis that increased plasma BHBA was due to increased rumen epithelial ketogenesis.

### Rumen papillae ultrastructure and histology

**Figure 2:**
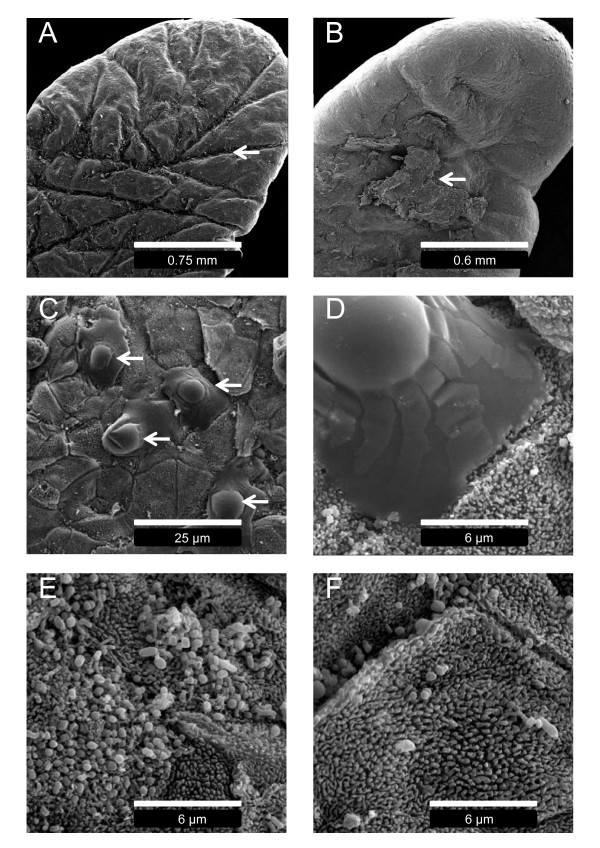
**Scanning electron micrographs of rumen papillae biopsied during the high forage and high grain diets**. A: rumen papillae biopsied during the high forage diet displaying large crevices (arrow). B: rumen papillae biopsied during the high grain diet with extensive sloughing of the stratum corneum (arrow). C&D: surface morphology of epithelium layer below sloughed corneum during high grain diet revealing non-differentiated keratinocytes (arrows). E: microbial colonization of the rumen epithelium during the high forage diet. F: microbial colonization of the rumen epithelium during the high grain diet.

To investigate changes in ruminal epithelium histology and ultrastructure, the biopsied and washed rumen papillae were prepared for scanning electron microscopy (SEM) and light microscopy. For SEM, the fixation protocol of rumen papillae was based upon methodology reported by Graham and Simmons [4]. Electron micrographs of the highly keratinized squamous rumen epithelium from the HF and HG diets are displayed in Figure 2. All papillae from the HF exhibited an intact keratinized stratum corneum with longer and deeper crevices throughout the surface of the papillae compared to the HG (Figure 2A). Rumen papillae from the HG showed evidence of extensive sloughing of the stratum corneum (Figure 2B). Non-differentiated keratinocytes with intact nuclei were discovered on the surface of the epithelium on the HG which is consistent with ruminal parakeratosis (Figure 2C&2D). Finally, microbial colonization of the epithelium was more apparent during the HF compared to the HG, and there was a greater number of microbial phenotypes present (Figure 2E&2F).

**Figure 3:**
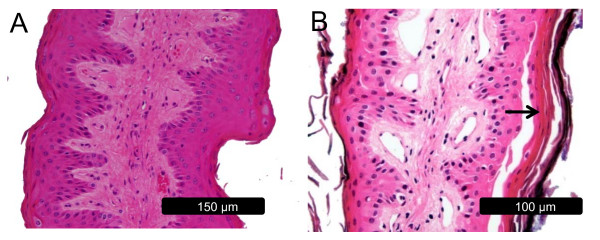
**Light micrographs of rumen papillae biopsied during the high forage and high grain diets**. A: rumen papillae from the high forage diet with an intact stratum corneum and granulosum. B: rumen papillae from the high grain diet displaying sloughing of the stratum corneum and demarcation of cells through the epithelial layers (arrow).

To further investigate the extent of epithelial sloughing, light microscopy slides were prepared using hematoxylin and eosin as described by Odongo et al. [11]. Cross-sections of papillae biopsied during the HF period (Figure 3A) displayed an intact stratum corneum and stratum granulosum. Sloughing of the stratum corneum was evident throughout the epithelium surface during acidosis. Below the stratum corneum the demarcation of the different strata became diffuse as cells from the stratum basale migrated luminally at an increased rate (Figure 3B). The adhesion between cells of the stratum corneum and stratum granulosum appeared to be compromised, as evidenced by large gaps between cells. Additionally, increased infiltration of the upper strata of the ruminal epithelium with lymphocyte-like cells was observed (micrographs not shown).

## Discussion

The objective of this case report was to describe how the structure and function of the rumen epithelium adapts during the initial stage of ruminal acidosis in a mature dairy cow. In this study, the acute form of ruminal acidosis (ruminal pH < 5.0) was induced rapidly and the four strata of the rumen epithelium was found to elicit an adaptive response to the acidic rumen environment. Although there may be limits to this case report as it is based upon one cow, the severe shift in dietary grain caused dramatic physiological responses enabling differences between the HF and HG period to be clearly detected.

The two levels of strata adjacent to the basal lamina are the stratum basale and stratum spinosum which have functional mitochondria contributing to the metabolic properties of the rumen epithelium [4]. In the fed state, the rumen epithelium synthesizes more ketone bodies, acetoacetate, and BHBA from the metabolism of butyrate and acetate, compared to synthesis by the liver [12]. In this study, the change from the HF to the HG diet may have increased epithelial synthesis of BHBA which, in turn, resulted in increased blood BHBA by 1.8 times while NEFA concentrations remained unchanged. To further support this observation, the expression of *HMGCS1*, a key regulatory enzyme of ketogenesis [13,14], was increased 1.6 times from the HF to the HG diet. Therefore, we propose that rumen epithelium ketogenesis increases during the initial stage of grain challenge due to increased substrate availability and as an attempt to metabolize, and thus remove, accumulated VFAs in the rumen.

The rumen epithelium acts as a protective layer between the rumen environment and portal circulation [4]. The epithelial layer adjacent to the stratum spinosum, termed the stratum granulosum, is characterized by tight gap junctions between cells which prevent diffusion of metabolites and microbes across the rumen epithelium. The stratum corneum, consisting of cornified keratinocytes, is adjacent to the lumen and acts as an additional protective barrier [3]. In this study, light microscopy and SEM provided clear evidence of sloughing of the stratum corneum during the HG diet. Furthermore, cellular adhesion in the stratum corneum and stratum spinosum appeared to be weakened after the period of acute ruminal acidosis. Rumen epithelium morphological alterations were prominent, considering the short duration of the induced acidosis. Compromised integrity of the ruminal epithelium can enable the transmigration of rumen microbes into portal circulation, leading to liver abscesses and an array of immunological responses [2]. One can speculate that the observed increases in lymphocyte-like cell infiltration into the upper layer of the ruminal epithelium during the feeding of the HG diet is a systemic immune response to this invasion.

Several experiments have shown that prolonged feeding of highly fermentable carbohydrates increases the number of cell layers in the stratum corneum leading to a condition termed parakeratosis [5]. In contrast, the micrographs in this study revealed extensive sloughing of the stratum corneum which decreased the number of cell layers. Interestingly, electron micrographs taken below the sloughing layers of the corneum exposed non-differentiated keratinocytes at different stages of nuclear disintegration, indicative of rapid keratinization [6]. This suggests that the initial response of the rumen epithelium to a dramatic increase in fermentable carbohydrates is sloughing of the stratum corneum followed by excessive keratinization of the epithelium which would lead to parakeratosis.

Microbial population fluctuations in the liquid phase of the rumen during ruminal acidosis have been characterized [2] yet changes in microbial populations associated with the microenvironment near and within the epithelium are not well documented [15]. In this study, microbial colonization of the epithelium and the apparent loss of adherent ruminal bacterial cell numbers between the HF and HG diet were dramatically different based upon SEM images of papillae surfaces. Although very little is known about epimural microbes, they are thought to perform essential roles in host-microbe interactions and rumen metabolism [15]. Since very few epimural microbes have been identified and their function is not fully recognized, understanding how microbial population dynamics and diversity influence the health and productivity of the cow during ruminal acidosis may be of future importance to understand the progression of this disease.

## Conclusion

To our knowledge, this case is the first to demonstrate such a rapid alteration of the ruminal epithelium in association with ruminal acidosis. We propose that dramatic shifts in rumen substrate cause the rumen epithelium to orchestrate a rapid and complex adaptive response to reorganize its structure and function to maintain whole-animal homeostasis. The rapid structural changes of the epithelium increase the animal's susceptibility to microbial infection and alter rumen metabolism and nutrient absorption [1]. Further research is needed in this area to develop strategies to enhance gastrointestinal adaptation in grain-fed ruminants, which will improve their digestive and absorptive capacity and result in improved animal productivity and well-being.

## Competing interests

The authors declare that they have no competing interests.

## Authors' contributions

MAS and BWM were responsible for conducting the study, data analysis and writing the manuscript. OA assisted with veterinary care along with the continuous rumen pH measurements and revisions of the manuscript. SEH assisted with sample collection and revisions of the manuscript. JC assisted in interpreting light and scanning electron micrographs and revisions of the manuscript.
